# *Upk3b* Is Dispensable for Development and Integrity of Urothelium and Mesothelium

**DOI:** 10.1371/journal.pone.0112112

**Published:** 2014-11-12

**Authors:** Carsten Rudat, Thomas Grieskamp, Christian Röhr, Rannar Airik, Christoph Wrede, Jan Hegermann, Bernhard G. Herrmann, Karin Schuster-Gossler, Andreas Kispert

**Affiliations:** 1 Institute of Molecular Biology, Medizinische Hochschule Hannover, Hannover, Germany; 2 Institute of Functional and Applied Anatomy, Medizinische Hochschule Hannover, Hannover, Germany; 3 Max Planck Institute for Molecular Genetics, Berlin, Germany; Heart Science Centre, Imperial College London, United Kingdom

## Abstract

The mesothelium, the lining of the coelomic cavities, and the urothelium, the inner lining of the urinary drainage system, are highly specialized epithelia that protect the underlying tissues from mechanical stress and seal them from the overlying fluid space. The development of these epithelia from simple precursors and the molecular characteristics of the mature tissues are poorly analyzed. Here, we show that *uroplakin 3B* (*Upk3b*), which encodes an integral membrane protein of the tetraspanin superfamily, is specifically expressed both in development as well as under homeostatic conditions in adult mice in the mesothelia of the body cavities, i.e., the epicardium and pericardium, the pleura and the peritoneum, and in the urothelium of the urinary tract. To analyze *Upk3b* function, we generated a *creERT2* knock-in allele by homologous recombination in embryonic stem cells. We show that *Upk3b^creERT2^* represents a null allele despite the lack of *creERT2* expression from the mutated locus. Morphological, histological and molecular analyses of *Upk3b*-deficient mice did not detect changes in differentiation or integrity of the urothelium and the mesothelia that cover internal organs. *Upk3b* is coexpressed with the closely related *Upk3a* gene in the urothelium but not in the mesothelium, leaving the possibility of a functional redundancy between the two genes in the urothelium only.

## Introduction

The inner lining of the urinary drainage system, i.e. of the renal pelvis, the ureter, the urinary bladder and the proximal part of the urethra, represents a highly specialized epithelium that is both flexible to accommodate the varying intraluminal pressure and tight to seal off the toxicity of the urinary fluid. A compelling structural feature of this urothelium is the presence of an elaborated surface barrier, which is composed of extracellular matrix as well as of integral membrane proteins. Members of the uroplakin protein family have been identified as crucial building units of this surface barrier that exhibits an almost crystalline organization (urothelial plaques) [1]. Uroplakins can be subdivided into three sub-groups that consist of Upk1a/Upk1b, Upk2 and Upk3a/Upk3b. The subgroups are distinguished by the number of transmembrane domains, by their glycosylation pattern and by the size of their cytoplasmic domain. Upk3a and Upk3b proteins are characterized by a single transmembrane domain, a glycosylated N-terminal luminal domain and a relatively large cytoplasmic domain, that may anchor the urothelial plaques to the cytoskeleton [2], [3]. Upk3a and Upk3b can form heterodimeric complexes with Upk1b, whereas Upk1a heterodimerizes with Upk2 [3], [4]. Functional analyses by gene targeting have uncovered a crucial role for *Upk2* and *Upk3a* in maintaining the impermeability of the urothelium [5], [6]. In both mutants renal dysfunction and hydronephrosis develop, i.e. fluid-mediated dilatation of the renal pelvis, most likely due to a reduction of urothelial plaques and urinary leakage. Analysis of urothelial function of *Upk1a*, *Upk1b* and *Upk3b* has not yet been performed.

A restriction of uroplakin function to the epithelial lining of the urinary tract was recently questioned by the finding in microarray analyses that *Upk3b* is enriched in peritoneal, pleural and pericardial mesothelia of mice. Subsequent *in situ* hybridization analysis confirmed *Upk3b* expression in the visceral mesothelium of the lung and heart, liver, spleen, intestine and testis in adult mice [7]. Mesothelia are monolayers of flattened squamous-like epithelial cells that line the pleural, pericardial and peritoneal cavities of the chest and the abdomen, respectively. They possess a parietal layer that covers the body wall and a visceral layer that covers the organ in the respective cavity. Adult mesothelia produce a lubricating fluid that allows the internal organs to slide over each other. During development individual cells of the mesothelia can undergo a mesenchymal transition and leave the epithelial integrity, invade the underlying space and differentiate into fibroblasts and smooth muscle cells [8]–[10]. The visceral pericardium, also known as epicardium, has been particularly well studied in recent years since it turned out to provide precursors for the cardiac fibroskeleton as well as smooth muscle cells of the coronary vasculature [11], [12]. Some studies reported endothelial and myocardial fates of epicardial cells [13], [14] although these findings were criticized for technical ambiguities [15], [16].

Identification of *Upk3b* expression in mesothelial tissues raises the interesting possibility that mesothelia and urothelia share structural features that may relate to efficient sealing of luminal spaces. To gain deeper insight into the role of *Upk3b* in these tissues, we wished to determine its expression both in development and homeostasis and analyze its functional requirement using gene-knock-out technology in mice.

Here, we provide a detailed expression analysis of *Upk3b* and show that *Upk3b-*deficiency does not affect the development and integrity of urothelium and mesothelium in mice.

## Materials and Methods

### Ethics statement

All animal work conducted for this study was approved by H. Hedrich and A. Bleich, former and present state heads of the animal facility at Medizinische Hochschule Hannover and was performed according to European and German legislation. The generation of the *Upk3b*-mutant mouse lines was approved by the Niedersächsisches Landesamt für Verbraucherschutz und Lebensmittelsicherheit (Permit Number: 33.9-42502-04-08/1518).

### Mice

For the generation of a *creERT2* knock-in allele of *Upk3b* a targeting vector was constructed to insert a *CreERT2* coding region (Addgene plasmid 14797) [17] followed by a *PGK-neo* cassette flanked by *loxP* sites [18] into the start codon of the *Upk3b* locus (Figure 3A). The integrity of the targeting vector was confirmed by restriction mapping and sequencing before the plasmid was linearized and electroporated into 129/SvCast ES cells. 24 h after electroporation, selection of transgenic clones was started by addition of 125 µg/ml G418 to the medium. Surviving colonies were expanded and subsequently screened for correct integration of the 3′-homology arm by PCR and for correct 5′-integration by Southern blot analysis. Three ES clones with verified homologous recombination of both arms were microinjected into CD1 mouse morulae. Chimeric males were mated to a *cre* deleter line (*Tbx3^tm1.1(cre)Vmc^*) [19] to remove the *PGK-neo* cassette. The double fluorescent *cre* reporter line (*Gt(ROSA)26Sor^tm4(ACTB-tdTomato,-EGFP)Luo^*, synonym: *R26^mTmG^*) [20] was obtained from the Jackson Laboratory (Bar Harbor, Maine, USA). All mice were maintained on an outbred (NMRI) background.

Embryos for expression analysis were derived form NMRI wild-type mice. The cell fate was analyzed in *Upk3b^creERT2/+^;Rosa26^mTmG/+^* embryos, obtained from matings of males double heterozygous for *Upk3b^creERT2^* and *R26^mTmG^* alleles and females heterozygous for *Upk3b^creERT2^*. In the latter case, tamoxifen (Sigma) was dissolved in ethanol at 100 mg/ml and then emulsified in corn oil (Sigma) to a final concentration of 12.5 mg/ml. 4 mg of tamoxifen were intraperitoneally injected into mice at gestation day 9.5. For timed pregnancies, vaginal plugs were checked in the morning after mating and noon was designated as embryonic day (E) 0.5. Female mice were sacrified by cervical dislocation. Embryos and organs were harvested in PBS, decapitated, fixed in 4% paraformaldehyde overnight and stored in 100% methanol at −20°C before further use. Genomic DNA prepared from yolk sacs or tail biopsies was used for genotyping by PCR.

### Histological analysis

For histological stainings embryos were fixed overnight in 4% paraformaldehyde, paraffin embedded, and sectioned to 4-µm. Sections were stained with hematoxylin and eosin following standard procedures.

### Electron Microscopy

All tissues were immersion-fixed in 150 mM HEPES, pH 7.35, containing 1.5% formaldehyde and 1.5% glutaraldehyde. Transmission Electron Microscopy: After incubation in 1% OsO_4_ (2 h at RT) and 4% uranyl acetate (overnight at 4°C) tissues were dehydrated in acetone and embedded in Epon. 50 nm sections were post-stained with uranyl acetate and lead citrate [21] and observed in a Morgagni TEM (FEI). Images were taken with a side mounted Veleta CCD camera. Scanning Electron Microscopy: Fixed tissues were dehydrated in acetone, critical point dried and gold sputtered. Images were taken in a Philips SEM 505 at 10 kV, using magnification/spot size of 2000x/50 nm or 8000x/20 nm.

### Immunofluorescence

For immunofluorescence analysis rabbit polyclonal antibody against GFP (1∶200 sc-8334, Santa Cruz), mouse monoclonal antibody against GFP (1∶200,11 814 460 001, Roche), rabbit polyclonal antibody against SM22alpha (Tagln) (1∶200, ab14106-100, Abcam), Fluorescein labeled GSL I – isolectin B4 (1∶100, FL-1101, VectorLabs), rat monoclonal antibody against endomucin (Emcn) (1∶2, a kind gift of D. Vestweber, MPI Münster; Germany), rabbit polyclonal against periostin (1∶200, ab14041, Abcam), mouse monoclonal antibody against alpha-Smooth muscle actin (Acta2), FITC-Conjugate (1∶200, F3777, Sigma), mouse monoclonal antibody against uroplakin1b (Upk1b) (1∶200, WH0007348M2, Sigma), rabbit polyclonal antibody against aquaporin2 (Aqp2) (1∶200, AB3274, Millipore), Fluorescein labeled *Lotus tetragonolobus* lectin (LTA) (FL 1321, VectorLabs) and mouse monoclonal antibody against MF20 (1∶200, Hybridoma Bank University of Iowa) were used as primary antibodies.

Biotinylated goat-anti-rabbit (Dianova, 1∶400), Alexa488 goat-anti-rabbit (Invitrogen, 1∶400), Alexa488 donkey-anti-mouse (Invitrogen A21202, 1∶400), Alexa-Fluor555 goat-anti-mouse (Invitrogen A-21424, 1∶400) and Alexa-Fluor555 goat-anti-rabbit (Invitrogen A-21428 1∶400) were used as secondary antibodies. Nuclei were stained with 4′,6-diamidino-2-phenylindole (DAPI) (Roth).

Immunofluorescence analysis was done on 4-µm paraffin sections. All sections were pressure cooked for 3 min in antigen unmasking solution (H-3300, Vector Laboratories Inc). The signal was amplified using the Tyramide Signal Amplification (TSA) system from Perkin-Elmer (NEL702001KT, Perkin Elmer LAS).

### *In situ* hybridization analysis

*In situ* hybridization analysis on 10-µm paraffin sections and on whole embryos with digoxigenin-labeled antisense riboprobes was performed as described [22].

### Semi-quantitative reverse transcription PCR

For semi-quantitative analysis of *Upk3b* expression three hearts each of wildtype, heterozygous and homozygous E12.5 embryos were pooled. RNA was extracted with RNAPure reagent (Peqlab) and DNaseI treated for 30 min at 37°C. RNA was reverse transcribed with RevertAid H-minus M-MuLV Reverse Transcriptase (Fermentas). For semi-quantitative PCR, the pools were adjusted to yield the same *Gapdh* intensity at the mid-logarithmic phase and the *Upk3b* PCR was performed on these pools. Quantification was performed with ImageJ [23].

### Image analysis

Sections were photographed using a Leica DM5000 microscope with Leica DFC300FX digital camera. Whole-mount specimens were photographed on Leica M420 with Fujix digital camera HC-300Z. Mosaic merge pictures of sections where documented using a Leica DMI6000B microscope with a Leica FC350FX digital camera. The Leica LAS AF 2.3 soft- ware was used to generate a mosaic merge of 7×5 single pictures, allowing 20% overlap of neighboring pictures. All images were processed in ImageJ [23] and Adobe Photoshop CS4.

## Results

### *Upk3b* is expressed in the urothelium and in mesothelial tissues during embryonic development

**Figure 1:**
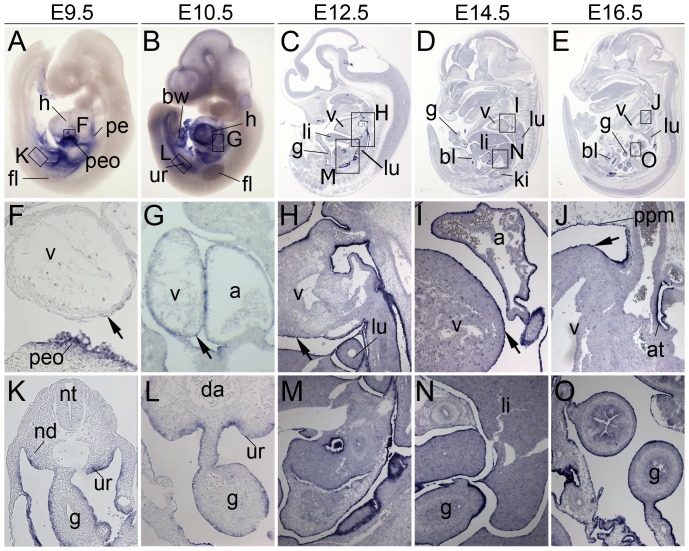
*Upk3b* expression in embryonic development. *In situ* hybridization analysis of *Upk3b* expression in whole wildtype embryos (A, B), on sagittal embryo sections (C–J and M–O) and on transverse embryo sections (K, L). (A–E) Overview images of embryos; anterior is up, dorsal is to the right. (F–O) Higher magnification images of the regions marked by open rectangles (in A–E). Stages are as indicated. Arrows point to the epicardium. a, atrium; at, epicardial covering of the dorsal wall of the great arterial trunks; bl, urinary bladder; bw, body wall; da, dorsal aorta; fl, fore limb bud; g, gut; h, heart; ki, kidney; li, liver; lu, lung; nd, nephric duct; nt, neural tube; ur, urogenital ridge; pe, pericardium of the dorsal wall of the pericardial coelom; peo, proepicardium; ppm, pleuropericardial membrane; v, ventricle.

To determine the expression pattern of *Upk3b* during embryonic development, we performed mRNA *in situ* hybridization analysis of whole embryos at E9.5 and E10.5 and of sections of E9.5 to E16.5 embryos (Figure 1). In E9.5 embryos expression of *Upk3b* was detected in the epithelial lining of the peritoneal cavity both in the parietal layer of the body wall and the visceral layer covering the urogenital ridge and the gut tube, in the proepicardium, in the pericardium of the dorsal wall of the pericardial coelom, and in single epicardial cells that were attached to the ventricular myocardium at this stage (Figure 1A, F and K). At E10.5, *Upk3b* was expressed in the contiguous epicardium in addition to all mesothelia of the peritoneal cavity (Figure 1B, G and L). At E12.5 and subsequent embryonic stages, all mesothelia (i.e. epicardium and pericardium, pleura, and peritoneum) expressed *Upk3b*. From E14.5 on, the epithelial lining of the developing urinary tract including the renal pelvis, the lumen of the bladder and the ureter was positive for *Upk3b* expression as well (Figure 1D–E, I–J and N–O).

### *Upk3b* is expressed in the pericardium and in the urothelium of adult mice

**Figure 2:**
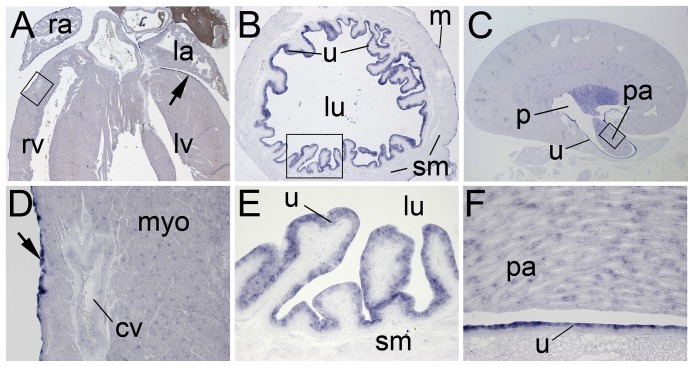
*Upk3b* expression in adult tissues. *In situ* hybridization analysis of *Upk3b* expression on sections of the adult heart (A, D), the urinary bladder (B, E) and the kidney (C, F). (A–C) Overview images of whole organ sections, (D–F) higher magnification images of the regions marked by open rectangles (in A–C). The arrow points to the epicardium. bl, urinary bladder; cv, coronary vessel; la, left atrium; lu, urinary bladder lumen; lv, left ventricle; m, urinary bladder mesothelium; p, renal pelvis; pa, renal papilla; ra, right atrium; rv, right ventricle; sm, smooth muscle layer, u, urothelium.

To test whether expression of *Upk3b* is maintained in adulthood, we performed *in situ* hybridization analysis on sections of organs obtained from 6-month old mice (Figure 2). In the heart *Upk3b* was confined to the epicardium that lined all chambers (Fig. 2A, D). In the urinary system, *Upk3b* expression was found in the multi-layered urothelium of the renal pelvis, the ureter and the bladder, in individual cells of the papillary collecting duct system and in the single-layered outer peritoneal lining of the bladder (Figure 2B, C, E, F).

### Generation of a *creERT2* knock-in allele of *Upk3b* by homologous recombination in ES cells

**Figure 3:**
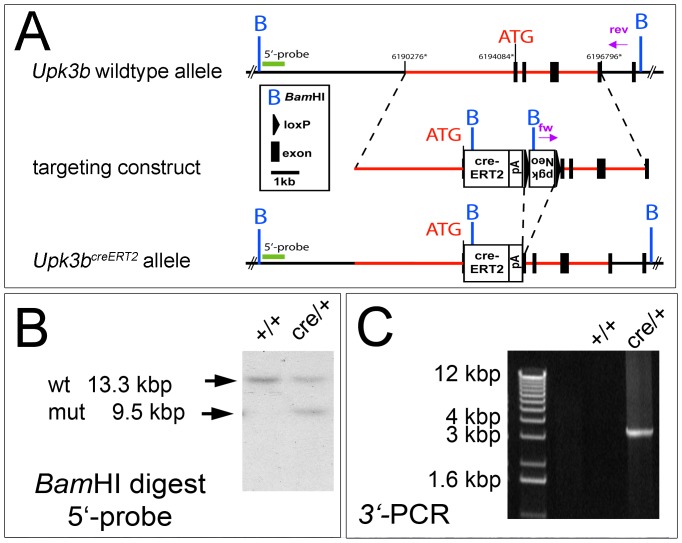
Generation and confirmation of a *creERT2* knock-in allele of *Upk3b*. (A) Scheme of the targeted insertion of a *creERT2* recombinase gene/*loxP*-flanked *neomycin* selection cassette in the *Upk3b* locus. Exons are shown in black, regions for homologous recombination in red. Screening for clones with correct integration of the *creERT2/neo* cassette was performed using a PCR for the 3′-region, primers are indicated in pink. (B) A *Bam*HI restriction fragment length polymorphism (RFLP) with the indicated 5′-probe was used to check for correct 5′-integration, wildtype (wt) and mutant (mut) bands are shown on the Southern blot. (C) A long-range PCR was used to verify the correct 3′-integration of the targeting vector by detection of a 3.5 kbp fragment in the mutant allele. ATG, transcriptional start codon; B, *Bam*HI; *creERT2*, *cre* recombinase fused to a triple mutant form of the human estrogen receptor expression cassette; kbp, kilo base pairs; *loxP*, locus of X-over P1; Neo, *neomycin* resistance gene; pA, polyadenylation signal; pgk, phosphoglycerate kinase I promoter.

**Figure 4:**
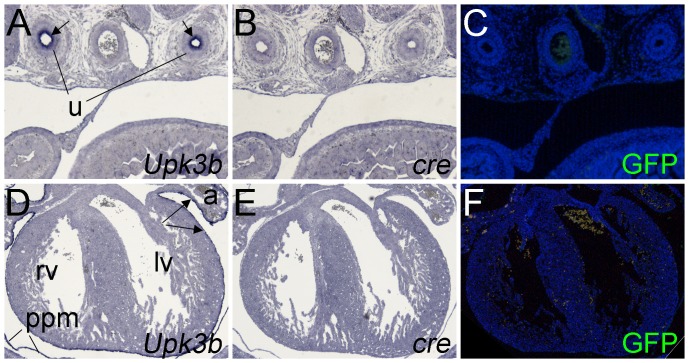
*Upk3b^creERT2/+^* mice neither express cre nor mediate recombination of *loxP*-flanked sequences in the ureter and in the epicardium. Pregnant mothers were injected with 2 mg tamoxifen at E9.5 and *Upk3b^creERT2/+^;Rosa26^mTmG/+^* embryos were analyzed at E15.5 by *in situ* hybridization for expression of *Upk3b* and *cre* on transverse sections of the ureter (A, B) and the heart (D, E). Immunofluorescence analysis of the lineage marker GFP was performed on transverse sections of the ureter (C) and the heart (F). Arrows (in A) point to the urothelium, arrows (in D) point to the epicardium. lv, left ventricle; ppm, pleuropericardial membrane; rv, right ventricle; u, ureter. Nuclei are counter-stained with 4′,6-diamidino-2-phenylindole.

To elucidate the role of *Upk3b* both in the development and in the maintenance of urothelial and mesothelial tissues in adulthood, we wished to generate an *Upk3*b knock-in allele allowing tamoxifen inducible expression of the *cre* recombinase gene under the control of endogenous *Upk3b* control elements (Figure 3). Mice with correct integration of a *creERT2* expression cassette in the *Upk3b* locus were obtained and subsequently tested for functionality of the creERT2 protein by injection of tamoxifen into pregnant *Upk3b^creERT2/+^;Rosa26^mTmG/+^* dams at E9.5. To our surprise, *in situ* hybridization did not detect expression of the *cre* transcript in the epicardium, the pericardium or ureteric urothelium of E15.5 embryos although expression of *Upk3b* was still easily detected in these organs (Figure 4A, B, D, E). Since this assay cannot unambiguously exclude the presence of low levels of *cre* expression, we additionally performed a reporter gene analysis using GFP immunofluorescence to test for recombination on neighboring sections. Expression of GFP was not detected in any of the analyzed tissues (Figure 4C, F). We, therefore, conclude that *creERT2* is not expressed in a correct manner from the *Upk3b^creERT2^* allele.

### Upk3b^creERT2/creERT2^ mice are Upk3b null mutants

**Figure 5:**
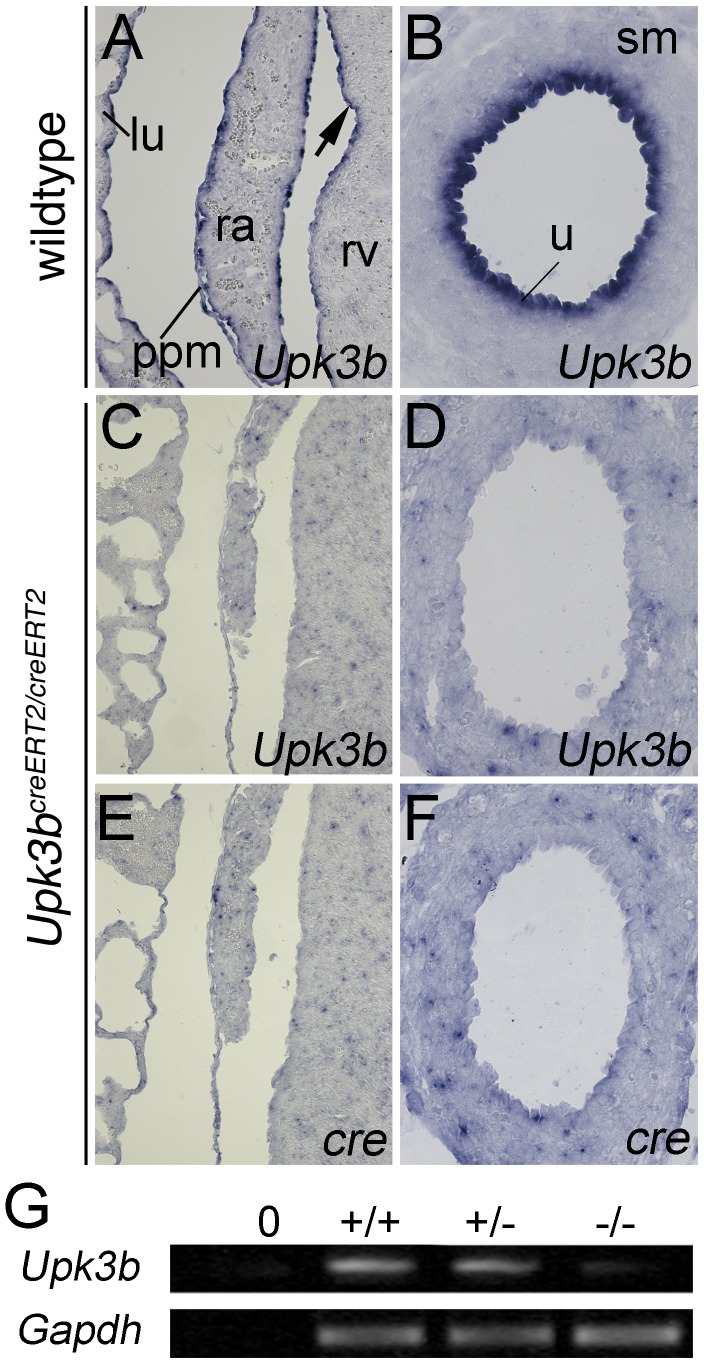
*Upk3b^creERT2/creERT2^* mice are *Upk3b* null mutants. *In situ* hybridization analysis for expression of *Upk3b* and *cre* on transverse sections of an E18.5 heart (A, C, E) and ureter (B, D, F) in wildtype and homozygous knock-out embryos. (G) Semiquantitative RT-PCR analysis of E12.5 isolated hearts derived from wildtype, heterozygous and knock-out (*Upk3b^creERT2/creERT2^*) animals. lu, lung; ppm, pleuropericardial membrane; ra, right atrium; rv, right ventricle; u, urothelium.

To test if *Upk3b* is deleted in the *Upk3b^creERT2^* allele, we performed *in situ* hybridization analysis for the *Upk3b*-3′-untranslated region on transverse sections of the heart and ureter in E18.5 embryos homozygous for the *Upk3b^creERT2^* allele. Expression of *Upk3b* was neither detected in mesothelia (e.g. lung, pleuropericardial membrane and epicardium) nor in the urothelium of the ureter (Figure 5A–F). Furthermore, semi-quantitative RT-PCR analysis of E12.5 isolated hearts derived from wildtype, heterozygous and homozygous mutant embryos confirmed the absence of *Upk3b* mRNA in *Upk3b^creERT2/creERT2^* embryos (Figure 5G). We conclude that *Upk3b^creERT2/creERT2^* mice represent *Upk3b* null mutants, and thus, can be analyzed for phenotypic consequences of loss of *Upk3b*.

### *Upk3b* is dispensable for development and integrity of the heart, the urinary bladder and the upper urogenital system

**Figure 6:**
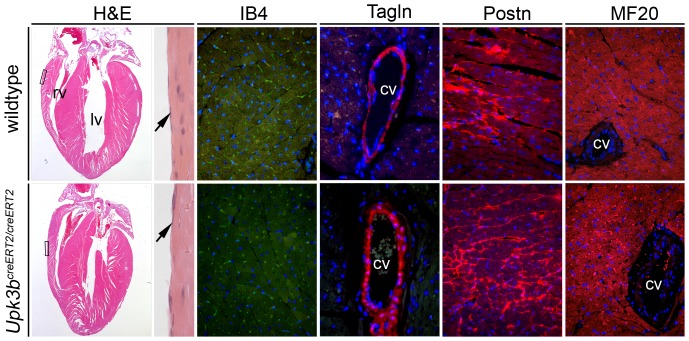
*Upk3b* is dispensable for normal heart formation. Hematoxylin and eosin staining (H&E) and immunofluorescence analysis of markers of capillary endothelia (IB4), cardiomyocytes (MF20), fibroblasts (Postn) and coronary smooth muscle cells (Tagln) on sections of 6-month old hearts of wildtype and homozygous knock-out mice. Magnified areas are indicated with rectangles. Arrows point to the epicardium. cv, coronary vessel; lv, left ventricle; rv, right ventricle. Nuclei are counter-stained with 4′,6-diamidino-2-phenylindole.

*Upk3b^creERT2/creERT2^* mice were born in the expected Mendelian ratio, reached sexual maturity and became fertile, and were unaltered in their behavior at 6-months of age. Morphologically, the mutants exhibited no differences in the appearance of internal organs of the chest and the abdomen at this age (data not shown). On histological sections the heart seemed unaffected; the ventricular wall thickness was normal and the integrity of the septa, valves and the epicardium was preserved (Figure 6).

Since epicardial cells give rise to the smooth muscle and fibroblast lineages, thus, contribute to the formation of the coronary vessels and fibrous skeleton of the heart, we analyzed by immunofluorescence of marker proteins the arrangement of vessels (isolectin-B4 endothelial staining), smooth muscle cells (transgelin (Tagln, also known as Sm22)) and of interstitial and perivascular fibroblasts (periostin (Postn)). Expression and distribution of these markers was indistinguishable between mutant and wildtype hearts, demonstrating that deletion of *Upk3b* is irrelevant for the integrity of the coronary vasculature and the cardiac fibrous skeleton at this level of resolution. Immunofluorescence analysis of the myocardial marker MF20 revealed a normal myocardium excluding changes of the trophic function of the mutant epicardium as well (Figure 6).

**Figure 7:**
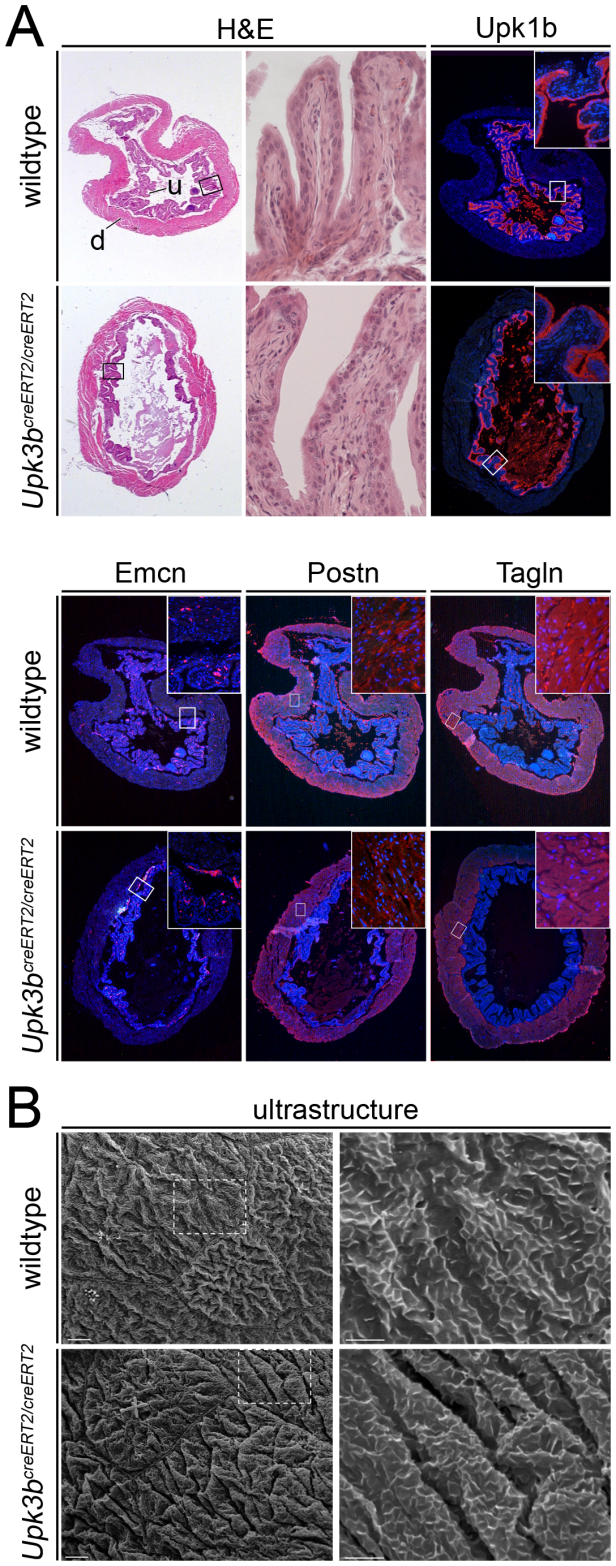
*Upk3b* is dispensable for normal urinary bladder formation. (A) Hematoxylin and eosin staining (H&E) and immunofluorescence analysis for markers of the urothelium (Upk1b), vessel endothelium (Emcn), fibroblasts (Postn) and smooth muscle cells (Tagln) on sections of the bladder of 6-month old wildtype and homozygous knock-out animals. (B) Scanning electron micrograph of the urothelial apical surface shows large polygonal superficial cells covered with microridges (arrows) in wildtype and homozygous knock-out animals. Magnified areas are indicated with rectangles. Bars represent 5-µm and 2-µm, respectively. d, detrusor; u, urothelium. Nuclei are counter-stained with 4′,6-diamidino-2-phenylindole.

The urinary bladder of 6-month old mutant mice appeared normal on histological sections; the urothelium and the detrusor muscle (the smooth muscle of the bladder) were in sound condition. Expression of urothelial (uroplakin1b (Upk1b)), endothelial (endomucin, Emcn), fibroblast (periostin, Postn) and smooth muscle markers (transgelin, Tagln, also known as Sm22)) was indistinguishable between mutant and wildtype mice (Figure 7A). In addition scanning electron micrographs of the apical surface of mutant bladder urothelium of 12-month old mutant mice showed umbrella cells covered with microridges indistinguishable from wildtype controls (Figure 7B), indicating that *Upk3b* is dispensable for normal urinary bladder formation and homeostasis.

**Figure 8:**
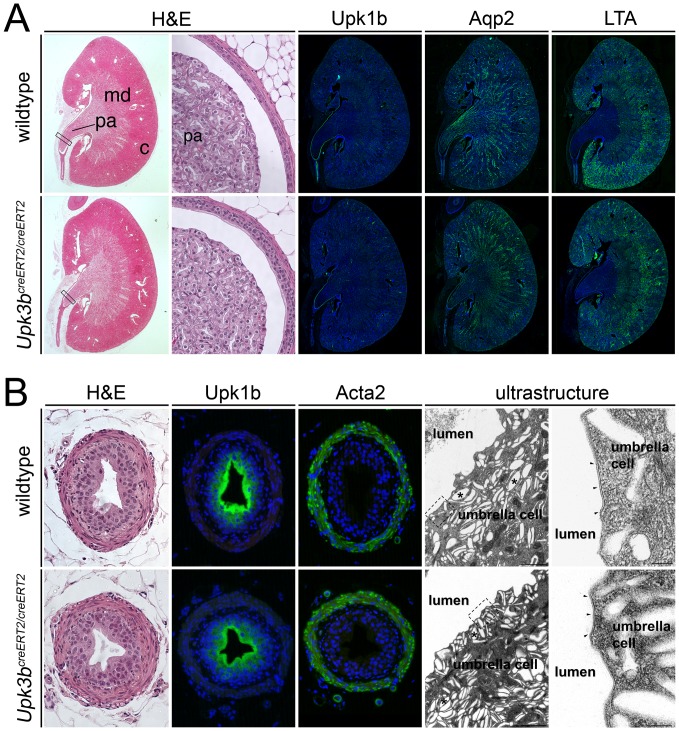
*Upk3b* is dispensable for normal kidney and ureter formation. (A) Hematoxylin and eosin staining (H&E) and immunofluorescence analysis for markers of the urothelium (Upk1b), collecting ducts (Aqp2) and proximal tubules (LTA) on sagittal sections of the kidney of 12-month old wildtype and homozygous knock-out animals. (B) Hematoxylin and eosin staining (H&E) and immunofluorescence analysis for markers of the urothelium (Upk1b) and smooth muscle cells (Acta2) and transmission electron micrographs on sections of the ureter of 12-month old wildtype and homozygous knock-out animals. TEM of mouse ureter umbrella cells showing fusiform cytoplasmic vesicles (asterisk) and apical plaques (arrowheads). Magnified areas are indicated with rectangles. Bars represent 1 µm and 100 nm, respectively. c, renal cortex; md, renal medulla; pa, renal papilla. Nuclei are counter-stained with 4′,6-diamidino-2-phenylindole.

Histological sections of adult kidneys showed normal zonation in renal cortex, medulla and papilla in *Upk3b*-deficient mice. Expression of Upk1b in the urothelium of the pelvis region, of aquaporin2 (Aqp2) in the collecting duct system and staining of proximal tubules with *Lotus tetragonolobus* agglutinin (LTA) was unchanged in the mutant (Figure 8A). We similarly did not detect histological, and molecular changes in the urothelium and the smooth muscle layer of the ureter in *Upk3b*-deficient mice. In addition, transmission electron micrographs of the apical surface of mutant ureter showed umbrella cells filled with fusiform vesicles and covered at the apical plasma membrane with urothelial plaques indistinguishable from wildtype controls, indicating that *Upk3b* is dispensable for normal ureter and kidney formation and homeostasis (Figure 8B).

### *Upk3a* is expressed in the urothelium but not in mesothelial tissues

**Figure 9:**
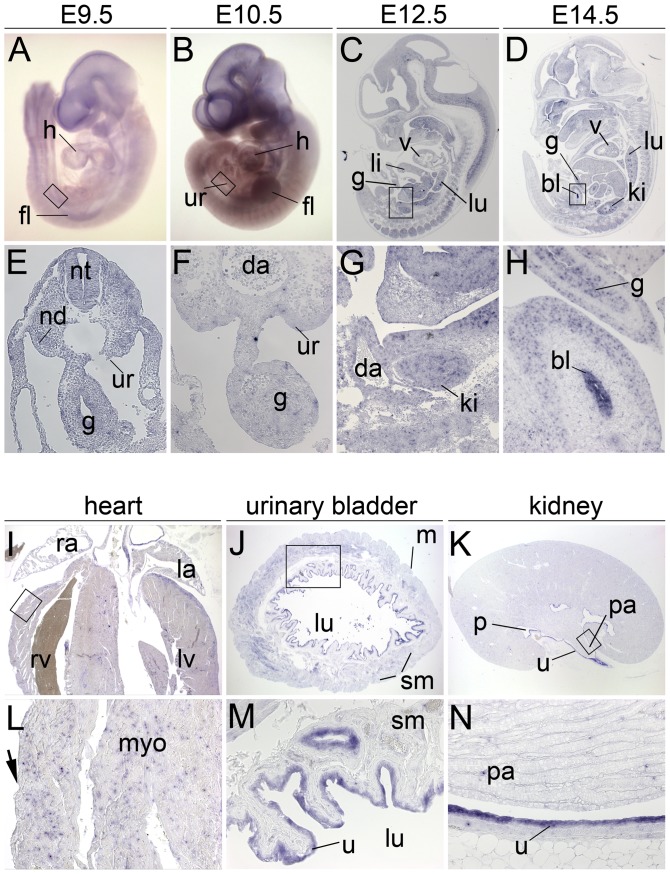
*Upk3a* expression in embryonic development and in adult tissues. *In situ* hybridization analysis of *Upk3a* expression in whole wildtype embryos (A, B), on sagittal embryo sections (C, D and G, H), on transverse embryo sections (E, F) and on sections of the adult heart (I, L), the urinary bladder (J, M) and the kidney (K, N). (A–D) Overview images of embryos; anterior is up, dorsal is to the right. (I–K) Overview images of whole organ sections; (E–H and L–N) higher magnification images of the regions marked by open rectangles (in A–D and I–K). Stages are as indicated. Arrows point to the epicardium. bl, urinary bladder; da, dorsal aorta; fl, fore limb bud; g, gut; h, heart; ki, kidney; la, left atrium; li, liver; lu, lung; lv, left ventricle; nd, nephric duct; nt, neural tube; p, renal pelvis; pa, renal papilla; ra, right atrium; rv, right ventricle; sm, smooth muscle layer; u, urothelium; ur, urogenital ridge; v, ventricle.

To test whether *Upk3a* might be able to compensate for the loss of *Upk3b* in certain tissues, we determined the expression pattern of *Upk3a* during embryonic development by mRNA *in situ* hybridization analysis of whole embryos at E9.5 and E10.5 and of sections of E9.5 to E14.5 embryos (Figure 9A–H). In E9.5 and E10.5 embryos, we did not detect expression of *Upk3a* (Figure 9A–B, E–F). At E12.5, we found weak neuronal expression in the central nervous system (Figure 9C). At E14.5, the urothelium of the renal pelvis, the bladder and the ureter was positive for *Upk3a* expression as well as a subpopulation of alveolar epithelial cells and the olfactory epithelium (Figure 9D, H). In 6-month old mice *Upk3a* expression was not detected in the epicardial layer of the heart but was found in the urothelium of the renal pelvis, the ureter and the bladder (Figure 9I–N).

## Discussion

### *Upk3b* is expressed in the urothelium and in mesothelial tissues during embryonic development and in adulthood

Our detailed expression analysis confirmed the urothelial presence of *Upk3b*. It additionally showed that *Upk3b* expression in the epithelium of the urinary tract precedes the begin of urine production around E16.0 in the kidney [24] compatible with the function of urothelial plaques to generate a permeability barrier against the toxic effects of the urine [1].

Our expression analysis for the first time identified *Upk3b* in all mesothelia from E9.5 to adulthood. The observed expression of *Upk3b* in the serosal mesothelium at E9.5 precedes the expression of the well established mesothelial marker *Wt1* [25]. This early presence of *Upk3b* underlines the importance of mesothelia in early gut morphogenesis and furthermore supports the model of an endogenous, resident rather than external progenitor pool of the serosal mesothelium [26]. Mesothelia are thought to be important for the protection of the underlying tissues, thus are likely to achieve a high degree of impermeability and flexibility at the same time. In analogy to the urothelium the occurrence of “mesothelial plaques” seems possible. To test this hypothesis, we performed additional expression analysis for the key components of the urothelial plaques *Upk1a*, *Upk2* and *Upk3a*. However, this analysis did not detect expression of any of these genes in mesothelial development (data not shown and Figure 9) making the formation of “mesothelial plaques” unlikely at this point. Nevertheless, luminal N-glycosylation of Upk3b protein [3] might account for the lubrication of mesothelial surfaces by binding of extracellular fluids. Furthermore, the potential interaction of Upk3b's cytoplasmic domain with the cytoskeleton may participate in the maintenance of the apical-basal polarity of mesothelial tissues.

### *Upk3b^creERT2^* represents a *Upk3b* null allele but does not allow cre-mediated recombination

Our targeting construct was designed to allow for tamoxifen-controllable mesothelial and urothelial expression of a creERT2 fusion protein. However, to our surprise we failed to detect expression and activity of this protein in mutant animals. At this point, we do not know the reasons that might have caused this problem. We assume that promotor accessibility might be affected by the knocked-in sequence, and silencing of the locus resulted. Furthermore, reduced transcript stability, due to reduced polyadenylation, altered 5′- and 3′-untranslated regions and overall translational activity are possible factors for the lack of creERT2 protein expression. Finally, potential mutations acquired during the ES cell culture leading to non-sense mediated decay, cannot be excluded. Nonetheless, *Upk3b* was no longer expressed from the mutant allele allowing the characterization of the phenotypic consequences of *Upk3b* loss in mice.

### *Upk3b* is dispensable for normal heart and urinary bladder formation

The specific expression of *Upk3b* in all mesothelia of the developing murine embryo pointed towards a possible role in the separation of the body cavities, in which growth of mesothelia is of crucial importance [27]. Differences in the separation of the chest and abdomen and of pleural, pericardial and peritoneal cavities as well as the appearance of internal organs were not detected in the mutants (data not shown), excluding an important function for *Upk3b* in the formation of mesothelia.

Mesothelial cells of the heart, lung, intestine and liver can give rise to vascular smooth muscle cells and fibroblasts [8], [10], [11], [25], [28]–[30]. Our analysis of the epicardium, the best studied mesothelial tissue in vertebrates, did neither detect changes in the mesenchymal transition of epicardial cells nor in the subsequent differentiation into smooth muscle cells and cardiac fibroblasts. Furthermore, we noticed that the ventricular myocardium was of normal thickness and the coronary vasculature was well-elaborated excluding both a cellular and trophic role of Upk3b in epicardial development.

Interestingly, adult mesothelial cells of the omentum and epicardium have been reported to contribute to vascular smooth muscle cell and fibroblast lineages under chronic and/or acute injury conditions [31], [32]. In peritoneal sclerosis, a submesothelial thickening of abdominal membranes [33] and in myocardial infarction new fibroblasts arise from the injured epicardium [34]. Often, these fibrotic conditions are additionally associated with inflammatory processes. As the molecular mechanisms underlying this regenerative capacity derive from the reactivation of embryonic gene programs [32], [35] that were unaffected in *Upk3b*-deficient embryos, we deem it unlikely that *Upk3b* is implicated in the regenerative capacity of adult mesothelia. However, future work should test a requirement for Upk3b in physiology and in pathological conditions in a more detailed fashion.

In urothelial plaques, Upk3b is present at low levels, amounting usually to less than 10% of Upk3a, the major plaque component. In *Upk3a*-deficient mice, urothelial plaques are present but smaller in size, *Upk3b* is up-regulated relative to other uroplakins [5], [3]. Furthermore, co-immunoprecipitation experiments showed specific binding of Upk3b to Upk1b, the binding partner of Upk3a in plaques. Together with our finding that *Upk3a* and *Upk3b* are coexpressed in the urothelium of the urinary tract and that the urinary tract appears normal in *Upk3b*-deficient mice, this suggests that *Upk3b* may act redundantly with *Upk3a* in the urothelium. Analysis of mice double mutant for *Upk3a* and *Upk3b* may address the combined function of both factors in the future.
